# The complete chloroplast genome sequence of *Bambusa lapidea* (Bambusodae)

**DOI:** 10.1080/23802359.2021.1939175

**Published:** 2021-06-21

**Authors:** Liguang Chen, Yangyang Zhang, Song Liu, Wenfeng Hu, Yongzhen Han, Ahsan Ul Haq, Yushan Zheng

**Affiliations:** a College of Forestry, Fujian Agriculture and Forestry University, Fuzhou, PR China; b Department of Forestry, Range Management and Wildlife, University of Agriculture Faisalabad, Pakistan

**Keywords:** *Bambusa lapidea*, plastid genome, Phylogeny, Bambusodae

## Abstract

*Bambusa lapidea* is primarily distributed in Guangdong, Guangxi, Sichuan, Yunnan, and Hong Kong in China, occurring on plains, lower hills, and wetlands on both sides of rivers and adjacent to villages. Therefore, we sequenced and reported the complete chloroplast genome of *B. lapidea* for the first time. The complete chloroplast genome sequence of *B. lapidea* was generated by *de novo* assembly using whole-genome next generation sequencing. The genome was 139,525 bp in total length, including a large next-copy (LSC) region of 83,034 bp, a small single-copy (SSC) region of 12,893 bp, a pair of invert repeats (IR) regions of 21,799 bp. The plastid genome contained 127 genes including 83 protein-coding genes, 36 tRNA genes, and eight rRNA genes. Phylogenetic analysis based on 14 chloroplast genomes indicates that *B. lapidea* is closely related to *B. arnhemica sinospinosa* and *B. teres* in Bambusodae.

*Bambusa lapidea* (https:/wcsp.science.kew.org/qsearch.do) is known for its outstanding fibrous raw materials with a large-scale clump bamboo species of high development and consumption value (Shi et al. 2009). The chloroplasts (cp) genome has a maternal inheritance and conserved structure that has been used to examine the developmental and phylogenetic relationships of plants (Wang et al. 2018). In this study, we identified the complete cp genome sequence of *B. lapidea* using Illumina sequencing data to explain the species' phylogenetic position and to do further evolutionary research. *B. lapidea* leaves specimens were taken from Fujian Province, China (University of Fujian Agriculture and Forestry, Bamboo Garden, Fuzhou:119°14′16″E, 26°5′7″ N) and quickly dried with silica gel for DNA extraction. The specimens were stored in the Bamboo Research Institute, Fujian Agriculture and Forestry University (contact person name: Tianyou He & Email: hetianyou@fafu.edu.cn) under the voucher number 101302). The improved CTAB method (Doyle and Doyle 1987) was used to extract DNA of the fresh leaf sample and its quantification was validated using Agarose gel electrophoresis, and Nanodrop concentration @500 bp randomly interrupted by the Covaris ultrasonic breaker for library construction. Approximately, 2.0 GB of raw data were generated with 150 bp paired-end read lengths. The Illumina High-throughput sequencing platform (HiSeq2500) data were filtered by the script in the NOVOPlasty (Dierckxsens et al. 2017). The complete cp genome of *Bambusa arnhemica* (GeneBank accession: KJ870989) as a reference and plastid genome of *B. lapidea* both were assembled by GetOrganelle pipe-line (https://github.com/Kinggerm/GetOrganelle), it can get the plastid-like reads. The cp genome annotation was assembled based on the comparison by Geneious v 11.1.5 (Biomatters Ltd, Auckland, New Zealand) (Kearse et al. 2012; Jin et al. 2018).

The final complete cp genome sequence of *B. Lapidea* has been submitted to GenBank under the accession number MW190087. Raw reads were deposited in the GenBank Sequence Read Archive (SRA PRJNA687881).The complete cp genome sequence of *B. Lapidea* was a circular shape of 139,525 bp in length, consisting of four distinct regions, such as a large single-copy (LSC) region of 83,034 bp, a small single-copy (SSC) region of 12,893 bp, and a pair of inverted repeats (IRa and IRb) regions of 21,799 bp each. Furthermore, the complete cp genome consisted of 127 genes, including 83 protein coding genes, 36 tRNA genes, eight rRNA genes, and 38.90% of GC content. Phylogenetic analyses including *B. lapidea*, 13 other Bambusodae species and one outgroup of Arundinarodae,were performed using complete cp genomes. All of them were downloaded from NCBI GenBank. The sequences were aligned by MAFFT v7.307 (Katoh and Standley 2013), and the phylogenetic tree was constructed by RAxML (Stamatakis 2014). In this study, the phylogenetic tree revealed that *B. lapidea* is closely related to *B. sinospinosa and B. teres* (Bambusodae) as illustrated in (Figure 1).

**Figure 1.**
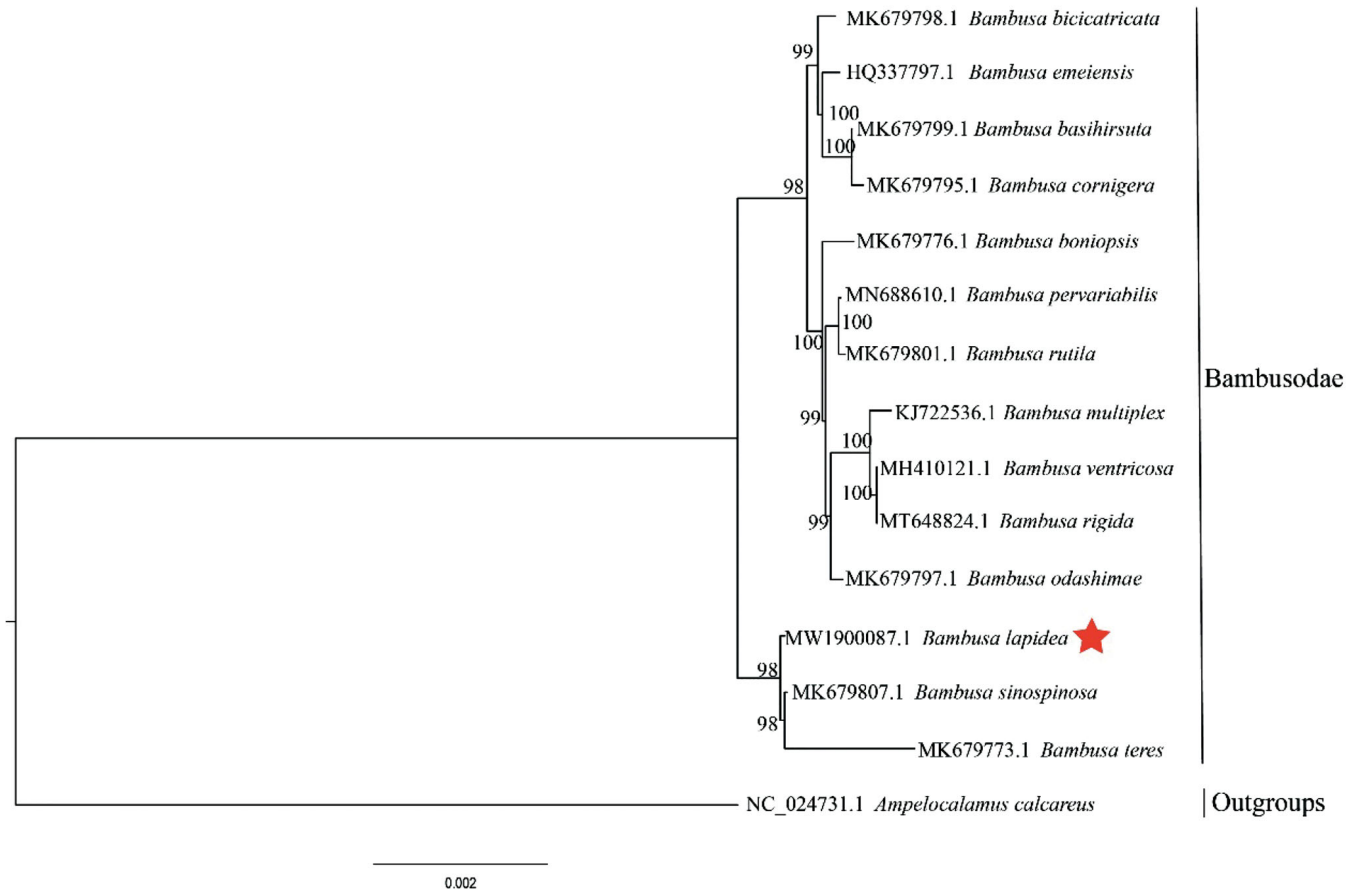
Maximum-likelihood phylogenetic tree based on complete cp genomes. Numbers close to each node are bootstrap support values.

## Data Availability

The genome sequence data that support the findings of this study are openly available in GeneBank of NCBI at (https://www.ncbi.nlm.nih.gov/) under the accession number MW190087. All high-throughput sequencing data files are available from the GenBank Sequence Read Archive (SRA) accession number: PRJNA687881.
